# Safety and Pharmacokinetics in Human Volunteers of Taniborbactam (VNRX-5133), a Novel Intravenous β-Lactamase Inhibitor

**DOI:** 10.1128/AAC.01053-21

**Published:** 2021-10-18

**Authors:** James A. Dowell, Daniel Dickerson, Tim Henkel

**Affiliations:** a Pharmacology Development Services, LLC, Collegeville, Pennsylvania, USA; b PRA Health Sciences, Lenexa, Kansas, USA; c Venatorx Pharmaceuticals, Inc., Malvern, Pennsylvania, USA

**Keywords:** taniborbactam, VNRX-5133, beta-lactamase inhibitor, drug safety, pharmacokinetics, first-in-human

## Abstract

Taniborbactam (formerly VNRX-5133), an investigational β-lactamase inhibitor active against both serine- and metallo-β-lactamases, is being developed in combination with cefepime to treat serious infections caused by multidrug-resistant Gram-negative bacteria. This first-in-human study evaluated the safety and pharmacokinetics of single and multiple doses of taniborbactam in healthy adult subjects. Single doses of 62.5 to 1,500 mg taniborbactam and multiple doses of 250 to 750 mg taniborbactam every 8 h (q8h) for 10 days were examined; all taniborbactam doses were administered as a 2-h intravenous infusion. No subjects experienced serious adverse events or discontinued treatment due to adverse events. The most common adverse event in both placebo- and taniborbactam-treated subjects was headache. The pharmacokinetics of taniborbactam were similar to the pharmacokinetics reported for cefepime. Taniborbactam demonstrated dose-proportional pharmacokinetics with low intersubject variability. Following single doses and with extended sampling, the mean terminal elimination half-life ranged from 3.4 to 5.8 h; however, the majority of exposure was characterized by an earlier phase with a half-life of about 2 h. Following multiple dosing, there was minimal accumulation of taniborbactam in plasma. At steady-state, approximately 90% of the administered dose of taniborbactam was recovered in urine as intact drug. There was no appreciable metabolism observed in either plasma or urine samples. (This study is registered at clinicaltrials.gov under registration number NCT02955459.)

## TEXT

The increasing incidence of infections caused by drug-resistant bacterial pathogens is a serious global health crisis and results in an estimated 700,000 deaths annually (1, 2). Of particular concern is the rapid emergence of β-lactamase-mediated resistance in Gram-negative pathogens, which renders the most commonly used class of antibiotics, β-lactams, ineffective (3). Recently approved β-lactamase inhibitors such as avibactam (4), vaborbactam (5, 6), and relebactam (7) are active against the serine β-lactamases of Ambler classes A and C and are variably active against class D but do not inhibit the Ambler class B metallo-β-lactamases (8).

Taniborbactam (formerly VNRX-5133) is an investigational, broad spectrum, cyclic boronate β-lactamase inhibitor that exhibits potent competitive inhibition of both Ambler classes A, C, and D serine-β-lactamases and the Ambler class B metallo-β-lactamases, such as Verona integron-encoded metallo-β-lactamases (VIM) and New Delhi metallo-β-lactamases (NDM) (9–12). In a global surveillance study, the combination of cefepime and taniborbactam (at a fixed concentration of 4 μg/ml) inhibited 99.7% of *Enterobacterales* isolates (*n* = 6,114 tested) and 94.5% of Pseudomonas aeruginosa isolates (*n* = 1,661 tested) at ≤8 μg/ml (13, 14). MIC results against these isolates confirmed that taniborbactam potentiates cefepime activity against *Enterobacterales* by at least 128-fold and against P. aeruginosa by 4-fold, as measured by the MIC_90_ (13, 14). In murine models, taniborbactam restored the *in vivo* efficacy of cefepime against cefepime-resistant β-lactamase-producing pathogens (15, 16). In a neutropenic murine thigh infection model using a human-simulated regimen of cefepime in combination with taniborbactam, the combination was shown to have potent *in vivo* activity against cefepime-resistant isolates, including serine-carbapenemase producers (17).

Cefepime-taniborbactam is being developed for the treatment of complicated urinary tract infections (cUTI) (clinicaltrials.gov under registration number NCT03840148) and for other serious infections in which multidrug-resistant Gram-negative pathogens occur (2, 18). The objective of this first-in-human study was to evaluate the safety and pharmacokinetics of single and multiple doses of taniborbactam in healthy adult volunteers.

## RESULTS

### Disposition and subject demographics.

In the single-ascending-dose (SAD) part of the study, 48 subjects were randomized, received a dose of study drug or placebo, and completed the study. In the multiple-ascending-dose (MAD) part, 36 subjects were enrolled, 1 subject in the placebo group withdrew, and 35 subjects completed the study. Subject demographic data are shown for the SAD and MAD parts in Tables 1 and 2, respectively. Distributions of subject sex and weight did vary across dose groups, with some groups having more males than females, with a corresponding increase in the group mean body weights relative to other groups.

**TABLE 1:** Subject demographics for the single-ascending-dose part

Parameter	Placebo (*n* = 12)	Taniborbactam dose					
		62.5 mg (*n* = 6)	125 mg (*n* = 6)	250 mg (*n* = 6)	500 mg (*n* = 6)	1,000 mg (*n* = 6)	1,500 mg (*n* = 6)
Sex [*n* (%)]							
Female	5 (41.7)	4 (66.7)	1 (16.7)	1 (16.7)	3 (50.0)	2 (33.3)	3 (50.0)
Male	7 (58.3)	2 (33.3)	5 (83.3)	5 (83.3)	3 (50.0)	4 (66.7)	3 (50.0)
Age (yrs) [mean (SD)]	32.3 (8.4)	27.0 (5.3)	31.8 (8.9)	28.7 (6.3)	27.3 (5.0)	35.0 (6.5)	34.5 (4.6)
Race [*n* (%)]							
White	7 (58.3)	4 (66.7)	2 (33.3)	2 (33.3)	3 (50.0)	1 (16.7)	5 (83.3)
Black or African American	5 (41.7)	2 (33.3)	4 (66.7)	2 (33.3)	3 (50.0)	4 (66.7)	1 (16.7)
Other	0	0	0	2 (33.3)	0	1 (16.7)	0
Wt (kg) [mean (SD)]	77.3 (9.5)	75.0 (16.6)	86.9 (16.9)	77.4 (9.1)	82.0 (10.0)	91.1 (14.4)	70.4 (17.2)

**TABLE 2:** Subject demographics for the multiple-ascending-dose part

Parameter	Placebo (*n* = 9)	Taniborbactam dosage		
		250 mg q8h (*n* = 9)	500 mg q8h (*n* = 9)	750 mg q8h (*n* = 9)
Sex [n (%)]				
Female	2 (22.2)	1 (11.1)	5 (55.6)	6 (66.7)
Male	7 (77.8)	8 (88.9)	4 (44.4)	3 (33.3)
Age (yrs) [mean (SD)]	30.9 (7.2)	32.2 (7.9)	26.7 (5.7)	30.6 (9.2)
Race [*n* (%)]				
White	4 (44.4)	6 (66.7)	5 (55.6)	4 (44.4)
Black or African American	4 (44.4)	2 (22.2)	4 (44.4)	5 (55.6)
Other	1 (11.1)	1 (11.1)	0	0
Wt (kg) [mean (SD)]	83.8 (13.5)	84.7 (13.8)	73.6 (12.1)	76.0 (18.8)

### Safety and tolerability.

There were no clinically significant changes in vital signs, clinical laboratory evaluations, ECGs, or physical examinations in either the SAD or MAD parts. The majority of adverse events were mild or moderate, and adverse event frequency and severity did not increase with increasing dose (Tables 3 and 4). There were no deaths, serious adverse events, or adverse events leading to discontinuation. The incidence of adverse events following treatment of taniborbactam was low and similar to placebo. In the SAD part, 4 subjects (33.3%) in the pooled placebo group and 7 subjects (19.4%) in the pooled taniborbactam group experienced treatment-emergent adverse events (TEAEs) (Table 3). Of these adverse events, only 2 were considered by the investigator to be related to treatment—1 subject in the taniborbactam 125-mg group (nausea) and 1 in the placebo group (headache). In the MAD part, 3 subjects (33.3%) treated with placebo and 8 subjects (29.6%) treated with taniborbactam experienced TEAEs (Table 4), of which 3 subjects in the placebo group (1 dry mouth, 2 headache) and 4 subjects in the pooled taniborbactam group (2 headache, 2 nausea, 1 dizziness, 1 diarrhea) had adverse events that the investigator considered to be treatment related.

**TABLE 3:** Incidence of treatment-emergent adverse events in the single-ascending-dose part

Adverse event^*a*^	No. (%) for each group							
	Placebo (*n* = 12)	Taniborbactam dose						
		62.5 mg (*n* = 6)	125 mg (*n* = 6)	250 mg (*n* = 6)	500 mg (*n* = 6)	1,000 mg (*n* = 6)	1,500 mg (*n* = 6)	Taniborbactam pooled (*n* = 36)
Subjects with at least one adverse event	4 (33.3)	1 (16.7)	1 (16.7)	3 (50.0)	1 (16.7)	0	1 (16.7)	7 (19.4)
Headache	2 (16.7)	0	0	0	1 (16.7)	0	0	1 (2.8)
Migraine	0	0	0	0	0	0	1 (16.7)	1 (2.8)
Abdominal pain	0	0	0	1 (16.7)	0	0	0	1 (2.8)
Nausea	0	0	1 (16.7)	0	0	0	0	1 (2.8)
Vomiting	0	0	0	1 (16.7)	0	0	0	1 (2.8)
Viral URI	1 (8.3)	0	0	1 (16.7)	0	0	0	1 (2.8)
Pharyngitis	1 (8.3)	0	0	0	0	0	0	0 (0.0)
Arthralgia	1 (8.3)	0	0	0	0	0	0	1 (2.8)
Back pain	0	1 (16.7)	0	0	0	0	0	1 (2.8)
Increased blood CPK	0	0	0	1 (16.7)	0	0	0	1 (2.8)
Increased hepatic enzyme	0	0	0	1 (16.7)	0	0	0	1 (2.8)

a CPK, creatine phosphokinase; URI, upper respiratory tract infection.

**TABLE 4:** Incidence of treatment-emergent adverse events in the multiple-ascending-dose part

Adverse event	No. (%) for each group				
	Placebo (*n* = 9)	Taniborbactam dosage^*a*^			Taniborbactam pooled (*n* = 27)
		250 mg q8h (*n* = 9)	500 mg q8h (*n* = 9)	750 mg q8h (*n* = 9)	
Subjects with at least one adverse event	3 (33.3)	2 (22.2)	4 (44.4)	2 (22.2)	8 (29.6)
Headache	2 (22.2)	0	1 (11.1)	2 (22.2)	3 (11.1)
Nausea	1 (11.1)	0	2 (22.2)	0	2 (7.4)
Constipation	0	1 (11.1)	1 (11.1)	0	2 (7.4)
Diarrhea	0	0	1 (11.1)	0	1 (3.7)
Dry mouth	1 (11.1)	0	0	0	0 (0.0)
Dizziness	0	0	1 (11.1)	0	1 (3.7)
Jaw pain	0	0	0	1 (11.1)	1 (3.7)
Phlebitis	0	1 (11.1)	0	0	1 (3.7)

a q8h, every 8 h.

Mean change from baseline QTcF (ΔQTcF) was very small in all taniborbactam SAD dose groups and generally followed the pattern observed in the pooled placebo group. The resulting mean placebo-corrected ΔQTcF (ΔΔQTcF) varied between –7.1 msec and 6.8 msec across all dose groups without relation to dose level or time of dosing. In the exposure-response analysis, the prespecified model with a treatment effect-specific intercept provided a good fit to the observed QTcF data. Based on this exposure-response analysis, a QT effect (ΔΔQTcF) above 10 msec can be excluded within the range of observed taniborbactam plasma concentrations, up to 80,000 ng/ml, corresponding to the maximum plasma concentration (*C*_max_) of the highest dose of taniborbactam studied, 1,500 mg. Additionally, taniborbactam at these doses did not have an effect on heart rate, T-wave morphology, or the PR and QRS interval.

### Pharmacokinetics. (i) Single-ascending-dose part.

**FIG 1:**
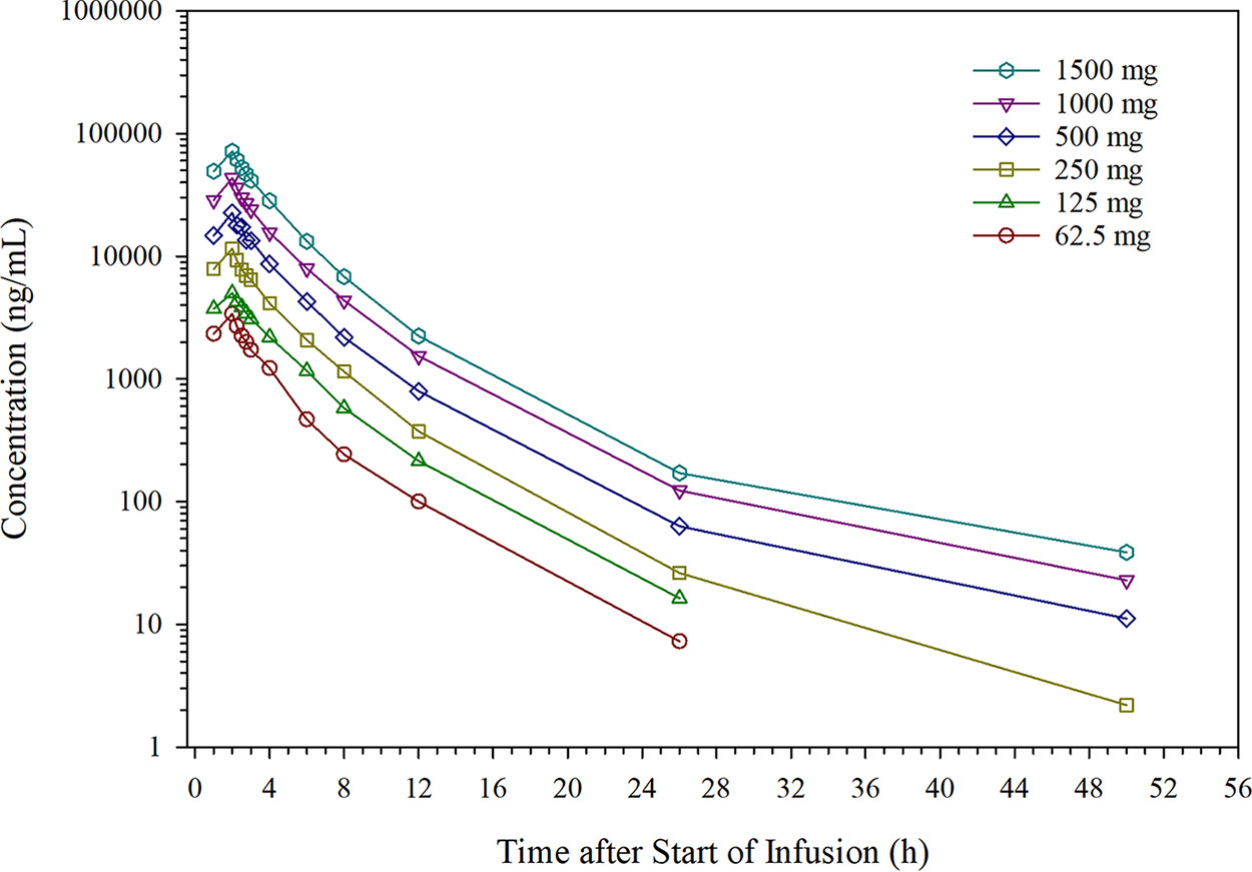
Mean taniborbactam plasma concentrations versus time following a single dose (single-ascending-dose part). Logarithmic concentration scale.

Individual taniborbactam plasma and urine concentration versus time profiles were quantifiable in all taniborbactam dose groups. In the SAD part, the maximum plasma concentration (*C*_max_) was observed immediately following the infusion, and concentrations decreased in a multiphasic manner for all dose groups (Fig. 1). Taniborbactam pharmacokinetic parameters for the SAD part are summarized in Table 5. The mean *C*_max_ increased with increasing dose from 3,398 ng/ml in the 62.5-mg group to 71,817 ng/ml in the 1,500-mg group. Taniborbactam exposure, as measured by the area under the plasma concentration versus time curve (AUC) extrapolated through infinity (AUC_inf_), increased with increasing dose from 11,284 to 263,422 h · ng/ml for the 62.5 and 1,500-mg-dose groups, respectively. Exposure following single doses was dose proportional across the range of doses studied, with slope estimates of 0.980 (90% confidence interval [CI], 0.956 to 1.004) and 0.986 (90% CI, 0.958 to 1.014) for *C*_max_ and AUC_inf_, respectively. The mean taniborbactam volume of distribution estimated using the terminal phase (*V_z_*) and total body clearance (CL) were consistent across dose groups, ranging from 28.4 to 54.0 liters and 5.6 to 6.5 liters/h across dose groups, respectively. The mean terminal elimination half-life (*t*_1/2_) ranged from 3.4 to 5.8 h across the dose groups. Higher-dose groups (500 to 1,500 mg) had measurable concentrations through the last collected sample for all subjects in the group, which allowed for better estimates of the terminal phase elimination rate (λ_Z_) and parameters dependent on λ_Z_. The *t*_1/2_ for subjects in higher dose groups were well estimated and similar (*t*_1/2_ ∼5.7 h). The majority of taniborbactam was recovered in the urine as intact drug. The estimated amount of taniborbactam excreted in urine (Ae) over a 48-h period after the single dose was approximately proportional across the examined range. The mean fractions of drug excreted unchanged in urine (Fe) following single doses of 500, 1,000, and 1,500 mg taniborbactam were 64.1%, 76.7%, and 66.6%, respectively. Renal clearance (CL_R_) was estimated using the Ae and AUC_inf_ and was shown to represent the majority of the overall CL. The mean CL_R_ was 3.9, 5.0, and 3.9 liters/h for single doses of 500, 1,000, and 1,500 mg taniborbactam, respectively. Intersubject variability within dose groups for most pharmacokinetic parameters was low.

**TABLE 5:** Summary statistics of taniborbactam pharmacokinetic parameters for single-ascending-dose part^*a*^*^,^*^*b*^

Parameter^*a*^	Taniborbactam dose					
	62.5 mg (*n* = 6)	125 mg (*n* = 6)	250 mg (*n* = 6)	500 mg (*n* = 6)	1,000 mg (*n* = 6)	1,500 mg (*n* = 6)
*C*_max_ (ng/ml)	3,398 (17.0)	5,023 (13.8)	11,533 (13.8)	22,650 (11.4)	43,317 (14.8)	71,817 (16.3)
*T*_max_ (h)	2.0 (2.0, 2.0)	2.0 (2.0, 2.0)	2.0 (2.0, 2.0)	2.0 (2.0, 2.0)	2.0 (2.0, 2.1)	2.0 (2.0, 2.0)
AUC_inf_ (h · ng/ml)	11,284 (13.3)	20,326 (13.7)	41,106 (19.0)	82,499 (9.2)	156,542 (15.7)	263,422 (17.8)
*V_z_* (liters)	28.4 (18.7)	30.2 (14.2)	34.6 (17.1)	50.2 (12.1)	54.0 (17.9)	49.4 (21.6)
CL (liters/h)	5.6 (14.1)	6.2 (12.6)	6.3 (17.5)	6.1 (9.0)	6.5 (15.7)	5.9 (19.2)
*t*_1/2_ (h)	3.5 (9.3)	3.4 (5.8)	4.0 (31.1)	5.7 (4.3)	5.7 (3.3)	5.8 (6.2)
Ae (mg)	ND^*c*^	ND	ND	320.6 (9.6)	766.9 (9.4)	998.7 (12.9)
Fe (%)	ND	ND	ND	64.1 (9.6)	76.7 (9.4)	66.6 (12.9)
CL_R_ (liters/h)	ND	ND	ND	3.9 (15.5)	5.0 (16.7)	3.9 (16.8)

a Ae, estimated amount of taniborbactam excreted in urine; AUC_inf_, area under the plasma concentration versus time curve extrapolated through infinity; *C*_max_, maximum plasma concentration; CL, total body clearance; CL_R_, renal clearance; *t*_1/2_, terminal elimination half-life; *T*_max_, time to maximum plasma concentration; *V_z_*, volume of distribution estimated using the terminal phase; Fe, fraction of drug excreted unchanged in urine.

b Statistics show arithmetic means (percent coefficient of variation). For *T*_max_, statistics show the median (range).

c Not determined for dose group.

**FIG 2:**
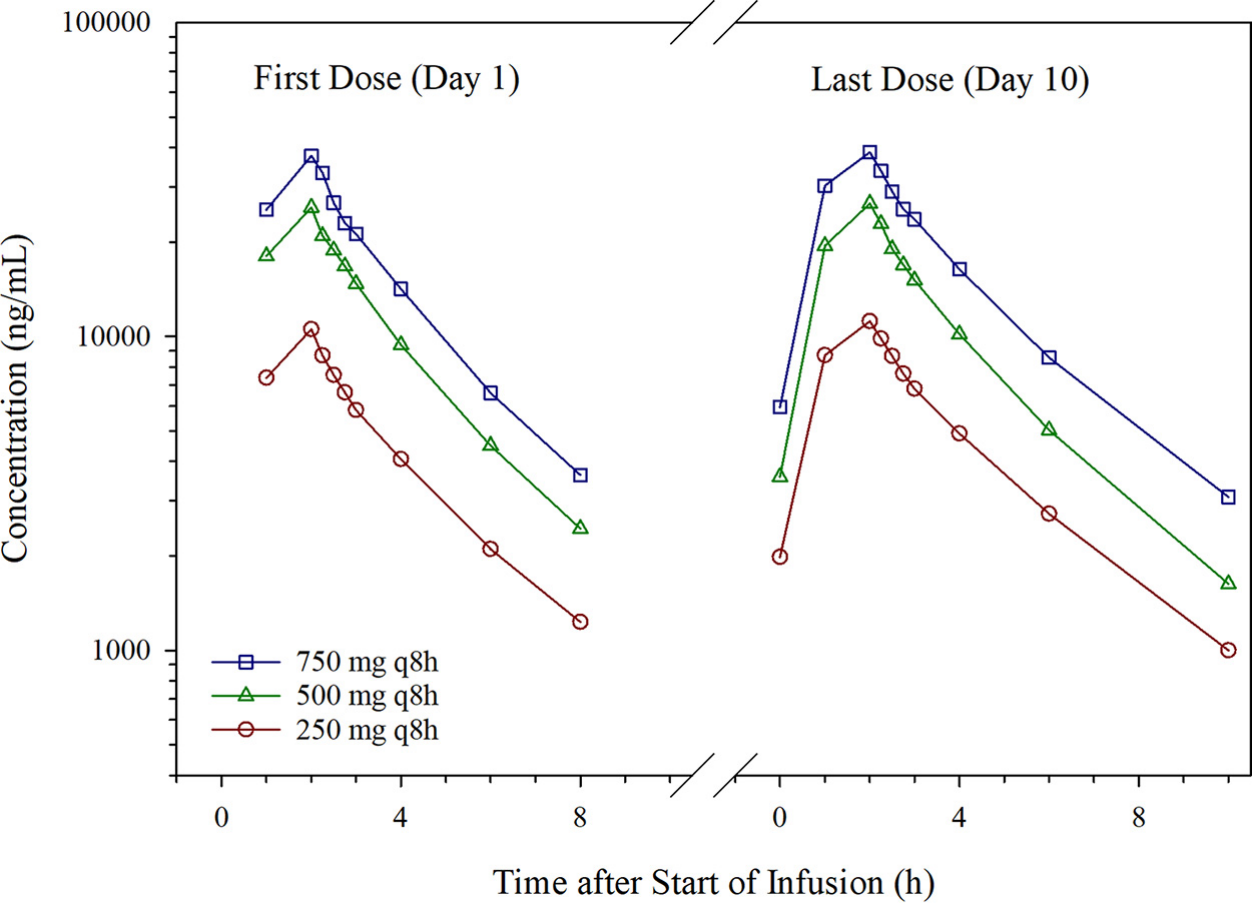
Mean taniborbactam plasma concentration versus time through the dosing interval (multiple-ascending-dose part). q8h, every 8 h. Logarithmic concentration scale.

**(ii) Multiple-ascending-dose part.** In the MAD part, pharmacokinetic profiles were collected on day 1 following the first dose and on day 10 following the last dose (total of 28 doses). As with the SAD pharmacokinetic profiles, the *C*_max_ was observed immediately following the infusion. Within the sampled dosing intervals (day 1 and day 10), taniborbactam plasma concentrations decreased in a monophasic manner (Fig. 2). Taniborbactam pharmacokinetic parameters for the MAD part are summarized in Table 6. Exposure increased with increasing dosage, and pharmacokinetic parameters were similar between the first and last dose, indicating minimal accumulation with multiple-dosing. The taniborbactam AUC through the dosing interval (AUC_tau_) mean ratios for the last dose compared to the first dose were 1.21, 1.09, and 1.17 for dosages of 250, 500, and 750 mg q8h, respectively. Taniborbactam exposure following multiple doses was dose proportional across the range of dosages studied, with slope estimates of 1.062 (90% CI, 0.998 to 1.126) and 1.021 (90% CI, 0.937 to 1.106) for the last dose *C*_max_ and AUC_tau_, respectively. Sampling after the last dose period allowed for estimates of the terminal elimination phase and terminal elimination-dependent pharmacokinetic parameters such as *V_z_*. Estimated distribution volumes in the MAD part were consistent across groups and fell within the range determined from the SAD part. The CL was estimated using the dose and AUC_tau_ and was an estimate of the CL at the assumed steady-state (CL_SS_). The mean CL_SS_ ranged from 5.6 to 6.2 liters/h across multiple dose groups. The *t*_1/2_ following the last dose ranged from 4.1 to 4.9 h across the dose groups. In the MAD part, urine was collected through the last dosing interval (day 10). The Fe through the 8-h dosing interval at the assumed steady state was 84.4, 92.4, and 92.3% of the dose for dosages of 250, 500, and 750 mg q8h, respectively. The CL_R_ for the MAD part was estimated using the Ae through the dosing interval and AUC_tau_ following the last dose at the assumed steady state (CL_R,SS_). The CL_R,SS_ accounted for the majority of the overall CL_SS_. As with the SAD part, intersubject variability within dosage groups for most pharmacokinetic parameters was low.

**TABLE 6:** Summary statistics of taniborbactam pharmacokinetic parameters for multiple-ascending-dose part^*a*^*^,^*^*b*^

Parameter	Dosing day	Taniborbactam dosage		
		250 mg q8h (*n* = 9)	500 mg q8h (*n* = 9)	750 mg q8h (*n* = 9)
*C*_max_ (ng/ml)	1	10,544 (18.3)	25,778 (15.2)	39,122 (23.7)
	10	11,188 (16.5)	26,533 (13.0)	38,778 (18.2)
*T*_max_ (h)	1	2.0 (2.0, 2.1)	2.0 (2.0, 2.1)	2.0 (2.0, 2.3)
	10	2.0 (2.0, 2.1)	2.0 (2.0, 2.3)	2.0 (2.0, 2.3)
AUC_tau_ (h · ng/ml)	1	34,447 (17.2)	81,890 (14.7)	119,140 (18.0)
	10	41,563 (15.1)	89,103 (13.8)	139,518 (21.6)
*V_z_* (liters)	10	36.0 (28.3)	39.8 (14.3)	37.4 (19.9)
CL_SS_ (liters/h)	10	6.2 (15.9)	5.7 (12.6)	5.6 (21.1)
*t*_1/2_ (h)	10	4.1 (29.3)	4.9 (13.3)	4.7 (15.4)
Ae (mg)^*c*^	10	211.0 (11.8)	462.2 (10.2)	692.2 (21.7)
Fe (%)^*c*^	10	84.4 (11.8)	92.4 (10.2)	92.3 (21.7)
CL_R,SS_ (liters/h)^*c*^	10	5.2 (17.3)	5.3 (14.2)	5.2 (28.6)

a Ae, estimated amount of taniborbactam excreted in urine; AUC_tau_, area under the plasma concentration versus time curve through the dosing interval; *C*_max_, maximum plasma concentration; CL_R,SS_, renal clearance at the assumed steady-state; CL_SS_, total body clearance at the assumed steady-state; *t*_1/2_, terminal elimination half-life; *T*_max_, time to maximum plasma concentration; *V_z_*, volume of distribution estimated using the terminal phase; Fe, fraction of drug excreted unchanged in urine.

b Statistics show arithmetic means (percent coefficient of variation). For *T*_max_, statistics show the median (range).

c Urine parameters are based on urine collected and amounts recovered through the dosing interval (8 h) at the assumed steady state.

**Metabolite characterization.** There were no significant taniborbactam biotransformation products associated with a detectable UV response in pooled plasma samples from either the SAD or MAD part of the study. In urine, unchanged taniborbactam represented greater than 94.0% of the UV response in pooled samples collected up to 48 h postdose in the SAD part and through 8 h post-steady-state dose in the MAD part.

## DISCUSSION

Taniborbactam is an investigational cyclic boronate-containing β-lactamase inhibitor that has no intrinsic antibacterial activity. However, due to its potent inhibitory activity against both serine- and metallo-β-lactamases, and especially against emerging VIM and NDM metallo-β-lactamases, the combination of taniborbactam with cefepime has the potential to address the unmet clinical need for the treatment of serious infections due to multidrug-resistant Gram-negative bacteria (9, 11, 19).

This first-in-human study has shown taniborbactam to be well tolerated with no safety concerns in healthy adult volunteers receiving single doses of up to 1,500 mg and multiple doses of up to 750 mg q8h for 10 days (2,250 mg/day). There were no serious adverse events in the study, and adverse events were seen with similar frequencies in both the taniborbactam and placebo treatment groups. There was no increased incidence of adverse events with increasing taniborbactam dose level or frequency. Taniborbactam at the single dose levels and respective concentrations examined, including supratherapeutic levels, showed no evidence of cardiodynamic effects, including changes in QTcF, heart rate and T-wave morphology or on the PR and QRS interval.

Traditionally, β-lactamase inhibitors are coformulated with a partner β-lactam antibiotic with consideration given to both inhibitor activity and similarities in pharmacokinetic properties (19). As observed in another clinical study, the pharmacokinetics of taniborbactam are consistent with those of cefepime, and the two drugs can be coadministered safely without the potential for a pharmacokinetic drug-drug interaction (20). This will allow for similar dosing of taniborbactam and cefepime. Taniborbactam systemic exposure was shown to be dose proportional with low intersubject variability observed within dose groups. Body weight did appear to describe variability in the pharmacokinetic parameters and was tested and included as a covariate in the dose proportionality analysis. The inclusion of this covariate in the statistical analysis was necessary due to the imbalance of sex within some of the dose groups. The *t*_1/2_ was well estimated in higher-dose groups in the SAD part when sampling was extended and the slower terminal phase could be observed above the assay lower limit of quantitation; however, the majority of exposure was characterized by an earlier phase with a shorter half-life of approximately 2 h. This shorter, predominant *t*_1/2_ was also observed in the monophasic pharmacokinetic profiles within dose intervals produced by multiple-dosing. Data from the study suggest that coadministration of taniborbactam is unlikely to result in clinically meaningful drug-drug interactions. At steady state, approximately 90% of the taniborbactam dose was recovered as intact drug in the urine, and no appreciable metabolism of the drug was observed in either plasma or urine samples. High urine concentrations of excreted unchanged taniborbactam may also be beneficial when partnered with an antibiotic such as cefepime, which is also extensively excreted in urine (21–23). The combination of taniborbactam and cefepime has the potential to be an effective treatment for cUTI caused by multidrug-resistant pathogens (24).

The safety and tolerability of taniborbactam were demonstrated in healthy volunteers in this study, and taniborbactam was shown to have a favorable pharmacokinetic profile that will enable coadministration with antibiotics such as cefepime. This study supports the continued development of taniborbactam in future clinical trials in combination with cefepime as a treatment for serious infections caused by multidrug-resistant Gram-negative pathogens.

## MATERIALS AND METHODS

### Study design.

This was a randomized, double-blind, placebo-controlled study of single- and multiple-ascending-doses of taniborbactam in healthy volunteers. The study was performed at a single site in the United States. The study protocol was reviewed and approved by an institutional review board, and the study was performed in accordance with the ethical principles that have their origin in the Declaration of Helsinki. Study conduct complied with the International Council for Harmonisation Guideline for Good Clinical Practice and applicable regulatory requirements. All subjects provided written informed consent prior to any study specific procedures.

Eligible subjects were randomized to receive either taniborbactam or placebo in a 3:1 ratio within dose cohorts in both the SAD and MAD parts of the study. Each dose cohort in the SAD part of the study enrolled 8 subjects, and each dose cohort in the MAD part of the study enrolled 12 subjects. In the SAD part, subjects received 62.5, 125, 250, 500, 1,000, or 1,500 mg taniborbactam as a 2-h intravenous (i.v.) infusion. In the MAD part, subjects received 2-h intravenous i.v. infusions of 250, 500, or 750 mg taniborbactam q8h over 10 days, ending after a final morning dose on day 10 (total of 28 doses).

All safety and available pharmacokinetic data for a completed dose cohort were reviewed by a safety review committee before making the decision to escalate to the next dose cohort. A sentinel dosing design feature was also used for the first SAD dose cohort, wherein 2 subjects were dosed initially, 1 with taniborbactam and 1 with placebo. After demonstration of appropriate safety and tolerability in the sentinel subjects, the remaining 6 subjects were dosed 72 h later. Escalated dosing in the MAD part of the study was initiated after the 1,000-mg taniborbactam SAD cohort was completed.

### Subjects.

Healthy men or nonpregnant women, 18 to 45 years of age with a body mass index of ≥18.5 kg/m^2^ and ≤32 kg/m^2^ were eligible to participate in the study. Subjects were excluded if they had a history of drug allergy, used tobacco or any medications for a chronic condition, had a history of or current substance abuse, had a positive drug or alcohol screen, or had positive screening tests for hepatitis B surface antigen, anti-hepatitis C virus antibodies, or anti-human immunodeficiency virus 1 and 2 antibodies. A subject could only be enrolled within 1 dose cohort.

### Safety assessments.

Safety was assessed based on the occurrence of adverse events and evaluation of laboratory tests (chemistry, hematology, urinalysis), physical examination, vital signs, and 12-lead electrocardiograms (ECGs) that were performed throughout the study. Safety was followed through 7 days postdose in the SAD part and followed through 7 days post-last dose in the MAD part.

ECG and pharmacokinetic data from the SAD part of the study were used to construct a concentration-QT effect model to assess potential QT effects. The primary cardiodynamic safety parameter was the corrected QT interval by Fredericia (QTcF). The potential impact of taniborbactam on other ECG intervals and T-wave morphology was also assessed. The ECG monitoring in the study was intensive, with continuous Holter 12-lead ECGs recorded on day 1. ECG data used in the modeling consisted of extracted replicates at time points that matched the pharmacokinetic data. The concentration-QT effect model included the entire single-dose range of between 62.5 mg and 1,500 mg taniborbactam.

### Pharmacokinetic assessments.

Blood samples for assay of taniborbactam in plasma were collected predose and at 1, 2, 2.25, 2.5, 2.75, 3, 4, 6, 8, 12, 26, and 50 h after the start of drug infusion in the SAD part. For the MAD part, blood samples to determine taniborbactam plasma concentrations were collected predose and at 1, 2, 2.25, 2.5, 2.75, 3, 4, 6, and 8 h following the first dose (day 1) and predose and at 1, 2, 2.25, 2.5, 2.75, 3, 4, 6, 10, 14, and 26 h following the last dose (day 10). Urine was collected predose and at intervals of 0 to 4, 4 to 8, 8 to 24, and 24 to 48 h after the start of the infusion in the SAD part for the higher-dose groups (500 to 1,500 mg taniborbactam). For the MAD part, urine was collected through the dosing interval following the last dose using collection intervals of 0 to 4 and 4 to 8 h after the start of the infusion. At the end of each urine collection interval, the volume of each urine collection was recorded and a sample was taken.

### Bioanalytical methods.

Plasma and urine samples were assayed for taniborbactam and screened for possible metabolites. Human dipotassium ethylenediaminetetraacetic acid (K2EDTA) plasma samples were processed using protein precipitation and assayed using ultra-high-performance liquid chromatography with tandem mass spectrometric detection (LC-MS/MS). Both low- and high-range methods were developed and validated for plasma samples, with calibration ranges of 5 to 5,000 ng/ml and 100 to 100,000 ng/ml, respectively. Acidified urine samples were processed using solid-phase extraction and assayed using a validated LC-MS/MS method with a calibration range of 5.0 to 5,000 ng/ml. For both matrices, separation was accomplished using a Waters Acquity HSS T3 column (Milford, MA) at 50°C, and gradient elution using 10 mM ammonium formate with 1% formic acid as mobile phase A and 50:50:1 acetonitrile:methanol:formic acid (vol/vol/vol) as mobile phase B. A triple quadrupole mass spectrometer (API Triple Quad 5500; AB Sciex, Framingham, MA) equipped with a turbo-ion spray set was used for detection in positive ion mode, and quantification was based on multiple reaction monitoring. The internal standard used in the assays was D_4_-taniborbactam.

For the low-range plasma assay method, accuracy ranged from –9.1% to –1.6% for the within-run bias and from –8.1% to –3.2% for the overall bias; precision ranged from 1.8% to 2.8%. For the high-range plasma assay method, within-run and overall bias accuracy ranged from –1.9% to 4.9% and 0.8% to 3.1%, respectively; precision ranged from 2.1% to 4.1%. For the urine assay method, within-run and overall bias accuracy ranged from –4.9% to 7.8% and –0.8% to 3.3%, respectively; precision ranged from 1.9% to 8.7%. Sample dilutions of 10-fold and 50-fold were also included in the assay validations. Demonstrated sample stability met the requirements of the sample storage used in the study.

To assess possible metabolites of taniborbactam, plasma and urine samples were pooled across subjects by time points, processed, and analyzed by ultra-high performance liquid chromatography tandem mass spectrometry with a quadrupole time-of-flight mass spectrometer, for acquisition of high-resolution accurate mass data, in positive mode with electrospray ionization, incorporating in-line UV detection with a photodiode array detector (Waters Acquity UPLC). The acquired chromatographic and mass spectral data were compared to analogous data obtained for the corresponding predose and placebo-dose samples to identify metabolites potentially associated with taniborbactam metabolism. Chromatographic peaks observed to be unique to the samples were subject to investigation by mass spectrometric techniques.

### Pharmacokinetic analysis methods.

Individual subject plasma and urine taniborbactam pharmacokinetic parameters were evaluated using noncompartmental analysis methods (Phoenix WinNonlin, Certara, Princeton, NJ). Single-dose pharmacokinetic parameters were determined in the SAD part, and both single-dose (day 1) and multiple-dose (day 10) pharmacokinetic parameters were determined in the MAD part. Estimated pharmacokinetic parameters included *C*_max_, time to *C*_max_ (*T*_max_), AUC_tau_, AUC_0–inf_, *t*_1/2_, CL, V_Z_, Ae, Fe, and CL_R_. The actual sample times were used to calculate the pharmacokinetic parameters. Calculation of λ_Z_ was based on best fit of concentrations in the observed terminal elimination phase of a profile, and statistical inclusion of any parameter based on λ_Z_ required the goodness of fit statistic (*R*^2^) to be greater than 0.8. Pharmacokinetic data were summarized using descriptive statistics by treatment. Dose proportionality based on *C*_max_ and AUC was determined using linear regression to calculate the slope and 90% CI and observing whether a slope of 1 was included in the confidence interval. The dose proportionality analysis included subject body weight as a covariate in the analysis.
